# Nucleotide identity and variability among different Pakistani hepatitis C virus isolates

**DOI:** 10.1186/1743-422X-6-130

**Published:** 2009-08-24

**Authors:** Muhammad Idrees, Sadia Butt, Zunaira Awan, Mahwish Aftab, Bushra Khubaib, Irshad-ur Rehman, Madiha Akram, Sobia Manzoor, Haji Akbar, Shazia Rafiqe, Sheikh Riazuddin

**Affiliations:** 1 National Centre of Excellence in Molecular Biology, 87-West Canal Bank Road Thokar Niaz Baig Lahore-53700, University of the Punjab Lahore, Pakistan

## Abstract

**Background:**

The variability within the hepatitis C virus (HCV) genome has formed the basis for several genotyping methods and used widely for HCV genotyping worldwide.

**Aim:**

The aim of the present study was to determine percent nucleotide identity and variability in HCV isolates prevalent in different geographical regions of Pakistan.

**Methods:**

Sequencing analysis of the 5'noncoding region (5'-NCR) of 100 HCV RNA-positive patients representing all the four provinces of Pakistan were carried out using ABI PRISM 3100 Genetic Analyzer.

**Results:**

The results showed that type 3 is the predominant genotypes circulating in Pakistan, with an overall prevalence of 50%. Types 1 and 4 viruses were 9% and 6% respectively. The overall nucleotide similarity among different Pakistani isolates was 92.50% ± 0.50%. Pakistani isolates from different areas showed 7.5% ± 0.50% nucleotide variability in 5'NCR region. The percent nucleotide identity (PNI) was 98.11% ± 0.50% within Pakistani type 1 sequences, 98.10% ± 0.60% for type 3 sequences, and 99.80% ± 0.20% for type 4 sequences. The PNI between different genotypes was 93.90% ± 0.20% for type 1 and type 3, 94.80% ± 0.12% for type 1 and type 4, and 94.40% ± 0.22% for type 3 and type 4.

**Conclusion:**

Genotype 3 is the most prevalent HCV genotype in Pakistan. Minimum and maximum percent nucleotide divergences were noted between genotype 1 and 4 and 1 and 3 respectively.

## Background

Hepatitis C virus (HCV) belongs to the family *Flaviviridae*, genus Hepacivirus and is responsible for the second most common cause of viral hepatitis [1]. Presently, nearly 8-10% of Pakistani population [2], 2% of the USA population and 3% people worldwide are HCV carriers [3]. HCV has a positive-sense genome of approximately 9.6 kb and is subject to high rates of mutational changes [4]. Genetic heterogeneity of HCV isolated from different geographical regions was documented and at least six major genotypes with a series of subtypes of HCV have been identified so far [5]. The relative prevalence of these genotypes varies among different geographic regions such as subtypes 1a, 1b, 2a, 2c and 3a account for more than 90% of the HCV infections in North and South America, Europe, Russia, China, Japan, Australia, New Zealand and India [6,7]. Type 4 is prevalent in Egypt, North Africa, Central Africa, and the Middle East; type 5 has been described in South Africa and type 6 is primarily found in Southeast Asia [8].

HCV variants studies have been made in the neighboring countries of Pakistan including India, Thailand, Vietnam, Indonesia and Burma and it is clear from all theses studies that type 1, type 2, type 3, and type 6 variants are prevalent in these areas [9-11]. From Pakistan few studies are available on the distribution of various hepatitis C virus genotypes [12,13] however; none contained information on percent nucleotide identity among different isolates and geographic variation in the prevalence of various HCV genotypes. Therefore; 5'NCR sequence analysis followed by phylogenetic analysis was used for identifying different HCV variants, subtypes and genotypes in chronic HCV patients belonging to different geographical regions of Pakistan.

## Methods

### Patients and samples

One Hundred serum samples from chronic HCV carriers showing HCV RNA positivity and representing the four different areas of Pakistan such as Punjab (East), North West Frontier Province (NWFP) (North-west), Sindh (South-east) and Balochistan (South-west) were included in the study. The isolates from Punjab (number of isolates [*n*] = 25); NWFP (*n *= 25); Sindh (*n *= 25); or Balochistan (*n *= 25); are designated as P, N, S, or B, respectively, to identify the origin of the samples. A printed questionnaire was completed by each participant before the blood sample was collected after written informed consent. The study protocol was approved by the Institutional Ethical Committee. The demographic characteristics of the sequenced patients are shown in Table 1.

**Table 1:** Demographic characteristics of patients (N = 100).

*Characteristics*	*Punjab*	NWFP*	*Sindh*	*Balochistan*	*Total (N = 100)*
*Sex-No. (%)*					
Male	13 (52)	15 (60)	11 (44)	18 (72)	57
Female	12 (48)	10 (40)	14 (56)	7 (28)	43
*Age range-years*	25-61	21-65	18-55	20-57	21-65
Mean age (Y) ± SD^‡^	40 ± 5.0	35 ± 7.0	47 ± 8.0	38 ± 9.8	43 ± 10.4
**Socio-economic Status**					
*No. (%)*					
Lower class	17 (68)	15 (60)	19 (76)	21 (84)	72
Middle class	08 (32)	10 (40)	06 (24)	04 (16)	28
**Educational level ***No. (%)*					
Middle/above school	21 (84)	18 (72)	14 (56)	06 (24)	59
No/Primary school	04 (16)	07 (28)	11 (44)	19 (76)	41
**Mode of contamination**					
*No. (%)*					
Known	20 (80)	21 (84)	18 (72)	20 (80)	79
Unknown	05 (20)	04 (16)	07 (28)	05 (20)	21
**History of previous Surgeries/dental procedure ***No. (%)*					
Yes	07(28)	03 (12)	06 (24)	03 (12)	19
No	18 (72)	22 (88)	19 (76)	22 (88)	81
**Injected antibiotics/vitamins with used needle ***No. (%)*					
Yes	12 (48)	06 (24)	04 (16)	04 (16)	53
No	13 (52)	19 (76)	21 (84)	21 (84)	47
**Blood transfusion/blood products ***No. (%)*					
Yes	02 (8)	01 (4)	00 (0)	01 (4)	06
No	23 (92)	24 (96)	25 (100)	24 (96)	94
*HCV RNA level*					
< 400,000 IU/mL^$^	16 (64)	11 (44)	13 (52)	09 (36)	49
>400,000 IU/mL	09 (36)	14 (56)	12 (48)	16 (64)	51
*Cirrhosis-No (%)*					
Present	03 (12)	02 (8)	05 (20)	2 (8)	12
Absent	22 (88)	23 (92)	20 (80)	23 (92)	88

^‡^Standard deviation

*NWFP, North West Frontier Province

^$^IU/mL, international units per milliliter

### HCV RNA extraction and RT-PCR

HCV RNA was extracted from 100 μl serum sample using Gentra (Puregene, Minneapolis, MN 55441 USA) RNA isolation Kit according to the procedure given in the kit protocol. cDNA was synthesized at 37°C for 50 minutes using 1 μM of outer anti-sense primer and single tube nested PCR was done for 285-bp 5'NCR gene as described previously (Idrees et al. 2008). The PCR products were analyzed on 2% agarose gel.

### Sequencing PCR of 5'UTR region

The purified DNA was used as templates for sequencing PCR in the Big-Dye Terminator cycle sequencing ready reaction kit (Applied Biosystems). Samples were analyzed on an automated sequencer (ABI PRISM 3100 genetic analyzer; Applied Biosystems). Products were sequenced from both strands to get consensus sequences. Placed the reaction tubes in thermal cycler (PE 2700, ABI) and set the volume to 20 μl. The samples were preheated at 96°C for one minute and then run 35 cycles with the following parameters: at 96°C for 10 seconds, 50°C for 5 seconds and 60°C for 4 minutes.

### Purifying extension sample electrophoresis

The extension products were purified using ethanol precipitation method as described in the manual. Re-hydrated the pellet in 15 μl formamide and mixed well by up/down pipetting. Kept at room temperature for 15 minutes in dark. Heat denatured at 95°C for 5 minutes in thermal cycler and immediately put on ice for 5 minutes. The sequenced samples with BigDye terminators were electrophoresed on ABI PRISM 3100 instrument that is equipped with required modules and dye set/primer files.

### Phylogenetic analysis

Pakistani isolates sequenced in the present study were aligned with the representative number of sequences for each major genotype and subtype selected from the GenBank database with the help of the Multalign program. Pairwise comparisons for percent nucleotide homology and evolutionary distance were made. The accession numbers of the prototype genotype sequences used to compare the 5' NC sequences were as follows: 1a, M62321; 1b, D90208; 2a, D00944; 2b, D01221; 2c, D10075; 3a, D14307; 3b, D11443; 3c, D16612; 4a, M84848; 4b, M84845; 4c, M84862; 4d, M84832; 4e, M84828; 4f, M84829; 5a, M84860; and 6a, M84827. The phylogenetic analysis of HCV isolates was performed with MEGA 3.0 software [14]. Jukes-Cantor algorithms were utilized, and phylogenetic trees were constructed by the neighbor-joining method. The reliability of different phylogenetic groupings was evaluated by using the bootstrap-resampling test from the MEGA program (1,000 bootstrap replications).

## Results

**Figure 1:**
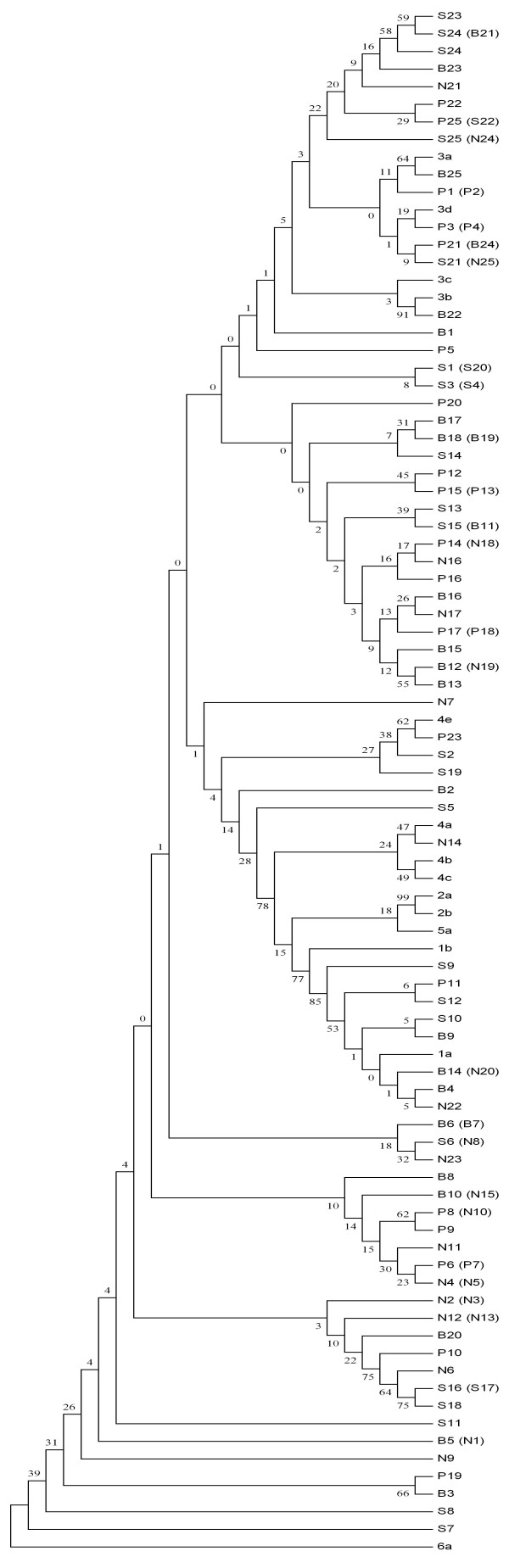
**Phylogenetic tree of HCV 5'UTR (nt 35 to 319) sequences of 100 HCV isolates**. To identify the origins of the samples, the isolates of HCV patients belonged to areas of Punjab, N.W.F.P., Sindh or Balochistan are designated as PP, PN, PS or PB respectively. Sequences for each major subtype were selected from GenBank database for analysis. The accession numbers of the reference sequences are as follows: M67463 (1a), D90208 (1b), AY051292 (1c), AF238485 (2a), D82034 (2b), D10075 54 (2c), AF046866 (3a), D11443 (3b), D16612 (3c), D16620 (3d), D16618 (3e), D16614 (3f), X91421 (3g), Y11604 (4a), M84845 (4b), M84862 (4c), M84832 (4d), M84828 (4e), 84829 (4f), M8486 (5a), and Y12083 (6a).

On the basis of phylogenetic analysis, the 100 Pakistani isolates were classified as follows: 50% type 3, 9% type 1 and 6% type 4. Thirty five isolates still remained untypable (Fig 1). It was not possible to differentiate between type 1b and 1c isolates further into different subtypes as both types clustered together. In the case of the type 3 isolates, there was a clear clustering of isolates into subtypes 3a and 3b but still there were isolates that were not clustering to any of the subtypes and these may be new subtypes. Frequency distributions of HCV genotypes were not similar in all the four regions of the country as can be seen in table 2. In the North-west region 60% of isolates were not typed (Table 2).

**Table 2:** HCV^‡ ^genotypes prevailing in Pakistan based on 5' NCR^$ ^sequence analysis (N = 100).

HCV Type	NWFP*	Punjab	Sindh	Balochistan	Total
1	2 (8%)	01 (4%)	03 (12%)	03 (12%)	9 (9%)
3	7 (28%)	17 (68%)	12 (48%)	14 (56%)	50 (50%)
4	01 (4%)	1 (4%)	3 (12%)	1 (4%)	6 (6%)
Not typed	15 (60%)	6 (24%)	7 (28%)	7 (28%)	35 (35%)
Total	25	25	25	25	100

^‡ ^HCV, hepatitis C virus

^$^NCR, Noncoding region

*NWFP, North West Frontier Province

The overall nucleotide similarity among these different Pakistani HCV sequenced isolates was 92.50% ± 0.50%. The percent nucleotide identity (PNI) was 98.11% ± 0.50% within Pakistani type 1 sequences, 98.10% ± 0.60% for type 3 sequences, and 99.80% ± 0.20% for type 4 sequences. The PNI between different genotypes was 93.90% ± 0.20% for type 1 and type 3, 94.80% ± 0.12% for type 1 and type 4, and 94.40% ± 0.22% for type 3 and type 4. There was a stretch of hypervariable region from nt: 83 to 171 in the 5'NCR of different HCV isolates. Pakistani isolates from different areas showed 7.5% ± 0.50% nucleotide variability in the sequenced 5'NCR region. The comparatively conserved stretch from nt 172 to 285 showed only 3.30% ± 1.06% variation. Minimum and maximum percent nucleotide divergences were noted between genotype 1 and 4 and 1 and 3. The sequence data of all the 100 sequences were submitted to GeneBank. The Accession Numbers provided for our nucleotide sequences by the GeneBank are from EF173931 to EF174030.

## Discussion

HCV is an RNA virus is with a high rate of genetic mutation and extensive genetic heterogeneity of HCV exists in infected individuals as a result HCV isolates are found as either a group of isolates with very closely related genomes quasispecies, or distinct groups genetically called genotypes. It is believed that the different HCV variants are relevant to epidemiological questions, vaccine development, clinical management, therapeutic decisions and strategies. Due to this vital importance of HCV variants, the present study was carried out to identifying different HCV genotypes from Pakistan in particular to find out variability among HCV isolates of the same and different genotypes. In the present study we were able to successfully sequence and classify an excellent percent of specimens. Several findings emerged from this study. The first finding is the observation that the direct sequencing of amplification products provides more detailed sequence information and could be useful in the detection of new viral types and subtypes. Further, it is clear from the results of the present study that direct sequencing of the 5'UTR fragment allows good discrimination among the HCV major types. Due to the high degree of conservation found within 5' NCR this approach is not able to completely differentiate between all subtypes.

It is further clear from the findings of the present study that in Pakistan, HCV genotypes show differing distributions in different geographic regions. HCV genotypes 1, 3 and 4 have been detected with genotype 3 being most frequently detected. Although genotype 4 is found almost exclusively in Middle East and western countries [15] this genotype is uncommon in our country. Unexpectedly genotype 4 was seen very rare in Balochistan that is attached to Iran in the South-west where genotype 4 is the second major type existing in that area [16]. Another important finding is the observation of the absence of genotype 2 in all the four different regions of the country though not surprising as from neighbor countries like India and Iran genotype 2 is reported very rare [7,16].

Next important finding of the present study is the isolation of many type 3 variants from Pakistan. The occurrence of many variants is not surprising because such type of variants have also been reported from neighboring countries particularly from India. The possibility of identifying more and more variants cannot be ruled out in the present situation of high prevalence of hepatitis C in this country. For this purpose, a study representing larger numbers of isolates from all provinces and community is required to generate countrywide data on HCV genotyping and variants.

## Conclusion

We conclude that (i) multiple HCV genotypes are prevalent in Pakistan with genotype 3a as the predominant HCV genotype circulating in Pakistan, (ii) 5'NCR sequence analysis is sufficient for the routine genotyping of isolates in clinical settings; however, sequencing is very expensive and needs special laboratory settings, expertise and this method is unable to detect more than one genotype if present in the patient, (iii) Minimum and maximum percent nucleotide divergences were noted between genotype 1 and 4 and 1 and 3 respectively.

## Abbreviations

HCV: hepatitis C virus; NCR: noncoding region; PNI: percent nucleotide identity; NWFP: North West frontier province; ABI: Applied Biosystem Inc.; RT-PCR: reverse transcriptase polymerase chain reaction; cDNA: complimentary DNA.

## Competing interests

The authors declare that they have no competing interests.

## Authors' contributions

SR conceived of the study, participated in its design and coordination and gave a critical view of manuscript writing. MI collected epidemiological data, sequenced and analyzed the data statistically. MI carried out the molecular genotyping assays. SR, SB, ZA, SM, MA, BK, HA and IR participated in data analysis. All the authors read and approved the final manuscript.
